# *Helicobacter*-induced gastric inflammation alters the properties of gastric tissue stem/progenitor cells

**DOI:** 10.1186/s12876-017-0706-6

**Published:** 2017-12-06

**Authors:** Wataru Shibata, Soichiro Sue, Sachiko Tsumura, Yasuaki Ishii, Takeshi Sato, Eri Kameta, Makoto Sugimori, Hiroaki Yamada, Hiroaki Kaneko, Tomohiko Sasaki, Tomohiro Ishii, Toshihide Tamura, Masaaki Kondo, Shin Maeda

**Affiliations:** 1 Department of Gastroenterology, Yokohama City University Graduate School of Medicine, Yokohama, Japan; 2 Division of Translational Research, Advanced Medical Research Center, Yokohama City University, Yokohama, Japan; 3 School of Medicine, Yokohama City University, Yokohama, Japan

**Keywords:** Helicobacter, Organoid, Intestinal metaplasia, SPEM; Spasmolytic polypeptide-expressing metaplasia, Cancer

## Abstract

**Background:**

Although *Helicobacter*-induced gastric inflammation is the major predisposing factor for gastric carcinogenesis, the precise mechanism by which chronic gastritis causes gastric cancer remains unclear. Intestinal and spasmolytic polypeptide-expressing metaplasia (SPEM) is considered as precancerous lesions, changes in epithelial tissue stem/progenitor cells after chronic inflammation has not been clarified yet. In this study, we utilized three-dimensional gastric epithelial cell culture systems that could form organoids, mimicking gastric epithelial layer, and characterized the changes in epithelial cells after chronic *Helicobacter felis* infection.

**Methods:**

We used three mice model; 1) long-term *H. felis* infection, 2) *H. felis* eradication, and 3) MNU chemical carcinogenesis model. We performed cRNA microarray analysis after organoid culture, and analyzed the effects of chronic gastric inflammation on tissue stem cells, by the size of organoid, mRNA expression profile and immunohistochemical analysis.

**Results:**

The number of organoids cultured from gastric epithelial cells was significantly higher in organoids isolated from *H. felis*-infected mice compared with those from uninfected gastric mucosa. Based on the mRNA expression profile, we found that possible stem cell markers such as *Cd44*, *Dclk1*, and genes associated with the intestinal phenotype, such as *Villin*, were increased in organoids isolated from *H. felis-*infected mucosa compared with the control. The upregulation of these genes were cancelled after *H. felis* eradication. In a xenograft model, tumors were generated only from organoids cultured from carcinogen-treated gastric mucosa, not from *H. felis* infected mucosa or control organoids.

**Conclusions:**

Our results suggested that, as a possible mechanism of gastric carcinogenesis, chronic inflammation induced by *H. felis* infection increased the number of tissue stem/progenitor cells and the expression of stem cell markers. These findings suggest that chronic inflammation may alter the direction of differentiation toward undifferentiated state and that drawbacks may enable cells to redifferentiate to intestinal metaplasia or neoplasia.

**Electronic supplementary material:**

The online version of this article (10.1186/s12876-017-0706-6) contains supplementary material, which is available to authorized users.

## Background

Gastric inflammation induced by CagA-positive *Helicobacter* infection is the major predisposing factor for gastric carcinogenesis [1–4]. Although the eradication of *Helicobacter pylori* (*H. pylori*) can reduce the risk of gastric cancer [5–7], considerable number of cancer has been found even after *H. pylori* eradication. Presence of certain type of metaplasia has been considered as the precursor of cancer cells and is associated with the risk of gastric cancer in human and mice [8–11]. Two types of metaplasia, are considered to be associated with gastric carcinogenesis in humans; intestinal metaplasia showed Muc2-positive intestinal goblet cells, whereas spasmolytic polypeptide-expressing metaplasia (SPEM) characterized TFF2-positive metaplasia through the transdifferetiation of chief cells [12]. In mouse model, chronic *Helicobacter* infection demonstrates only SPEM without progressing into intestinal metaplasia, but SPEM began to express intestinal phenotype after the long-term chronic inflammation [13].

Recently, a cancer stem/initiating cell concept was proposed to explain cancer development [14], and targeting cancer stem/initiating cells is a novel cancer-treatment strategy [15]. The cell surface marker CD44 is expressed in gastric cancer cells, and targeting of which may eliminate cancer cells resistant to radiation or chemotherapy [16–18]. Although the origin of cancer cells remains debated, cancer stem/initiating cells might be derived from tissue stem/progenitor cells due to the similar characteristics of both cells [19]. Lineage tracing studies have demonstrated several markers, such as *Villin, Lgr5, Sox2,* or *Troy* as candidate stem cell markers [20–24]. However, whether these markers could also cause gastric metaplasia, or gastric cancer, and also function as markers of stem cells in stomach remains to be elucidated.

To analyze the effects of chronic gastric inflammation on tissue stem cells and to examine the relationship between stem cells and carcinogenesis, we applied 3D gastric organoid culture systems, by which we could characterize the primary epithelial cells in vitro, tracking those cells for a long period of time and characterizing their properties without considering other interstitial cells [22, 25–27]. Here, we presented comprehensive molecular characteristics of gastric epithelial cells following chronic inflammation by using gastric organoid culture system combined with in vivo study, and showed that chronic inflammation induced by *Helicobacter* infection increased the number of tissue stem/progenitor cells, which acquired intestinal properties, and would contribute to gastric carcinogenesis.

## Methods

### Mouse model

**Fig. 1:**
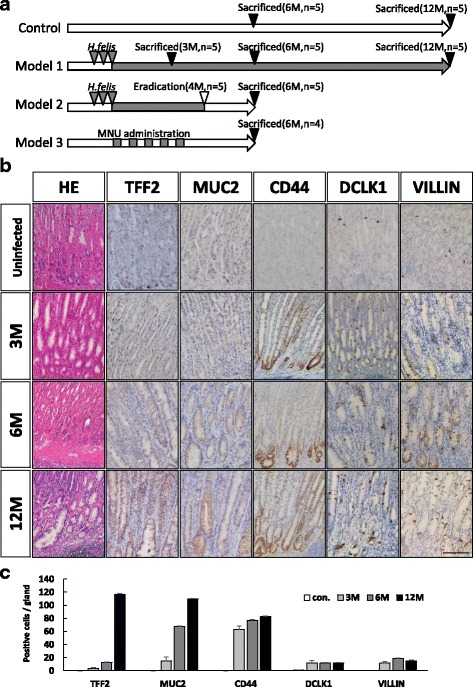
Increased expression of known gastric tissue stem/progenitor markers. **a** Schema of experimental model used in current study. **b** Representative photographs of hematoxylin and eosin (H&E) staining and immunohistochemical analyses of TFF2, MUC2, CD44, DCLK1, and VILLIN in stomachs infected with *Helicobacter felis* and uninfected control (original magnification, ×200, scale bar; 100 μm). **c** The number of positive cells for each antibody per gland

This research was approved by Institutional Animal Experiment Committee at Yokohama City University (Approved#; F-A-14-043). C57BL6/J wild-type mice were obtained from CLEA Japan Inc. (Tokyo, Japan). Male mice aged ≥8 weeks were used in the chronic gastritis model induced by *Helicobacter* infection [28]. Mice were sacrificed at 3, 6, and 12-month post infection, and the stomachs were removed and subjected to histological analysis or mRNA expression analysis (*n* = 5 per each time point, Model 1 in Fig. 1a). For eradication study (Model 2 in Fig. 1a), we used 5 mice as continuous infected group and 5 mice as eradicated group. Gastric epithelial cells from corpus area were used for organoid culture unless otherwise indicated [22]. We further investigated the tumorigenic activity of the organoids from *Helicobacter*-infected or chemical carcinogen N-methyl-N-nitroso-urea (MNU)-treated mucosa by injecting them into immune-deficient NOD/SCID (NOD.CB17-*Prkdc* ^*scid*^/J) mice purchased from Charles River Laboratories International, Inc. (Wilmington, MA, USA) (*n* = 4 per group). In MNU model, male 6-week-old wild-type mice were treated with water containing 240 ppm MNU (Sigma, St. Louis, MO, USA) in alternate weeks for a total of 5 weeks, as described previously [29]. Mice were sacrificed at 4 weeks after the final MNU dosing, and then isolated gastric epithelial cells from antrum area for subsequent organoid culture (Model 3 in Fig. 1a). Because MNU is a chemical carcinogen in the mouse gastric antrum, we used this model as a positive control to assess tumorigenicity. We first performed 3D organoid culture as described above, from mice either infected with *H. felis*, treated with MNU, or untreated control (Model 1 and 3 in Fig. 1a). After 4 days of culture, each group of organoids was injected subcutaneously into mice and the tumorigenicity and histological changes were analyzed after 2 months.

### Infection and eradication of *H. felis*

We used *Helicobacter felis* (*H. felis,* strain ATCC 49179) [30]. In brief, *H. felis* was cultured in trypticase soy broth at a titer of 1 × 10^7^ CFU/ml. The bacterial suspension was stored at −80 degree until use. 10 wild-type male mice aged 8 weeks were infected with 0.25 ml of *H. felis* (concentration; 1.0 × 10^7^ CFU/ml) suspension by oral gavage 3 times in a week. Four months after *H. felis* infection, mice were divided into two groups (5 mice each): one for continuous infection (Model 1 in Fig. 1) and the other for which *H. felis* was eradicated 2 months before sacrifice (Model 2 in Fig. 1) using a cocktail of antibiotics with tetracycline HCl (0.5 mg/30 g mouse/day), metronidazole (0.675 mg per 30 g mouse/day), and bismuth subsalicylate (0.185 mg per 30 g mouse/day) dissolved or suspended in a total volume of 500 μL sterile water, as described previously [31]. The cocktail was administered orally 5 days a week for 14 days. Eradication of *H. felis* was confirmed by histopathology and real-time PCR for *H. felis flaB* gene using gastric specimens as described previously [31]. We used 4 mice of 12 months’ post infection to analyze cRNA expression by microarray, and total 15 mice for immunohistochemical study (5 mice at 3 months, 5 mice at 6 months, and 5 mice at 12 months). Appropriate number of uninfected mice was used as experimental controls.

### 3-dimentional organoid culture of gastric epithelial cells

We cultured gastric epithelial cells in Matrigel in serum-free media for 3D organoid culture as described previously [32, 33]. Briefly, mouse gastric corpus was removed and cut into approximately 1-mm^2^ pieces. The gastric epithelial tissues were washed in ice-cold phosphate-buffered saline (PBS) three times. Next, the tissues were incubated in ice-cold PBS containing 10 mM EDTA at 4 °C for 3 h. After incubation, the tissue samples in PBS were vigorously shaken and centrifuged, and then the sediments were enriched in gastric glands for subsequent experiments. Isolated gastric glands were seeded on pre-warmed Matrigel plates (24 wells) supplemented with B27, N2, N-acetylcysteine (final 0.5 mM, Invitrogen, Carlsbad, CA, USA), and gastrin (10 nM; Sigma-Aldrich) containing growth factors (50 ng/ml epidermal growth factor [Peprotech, Rocky Hill, NJ, USA], 1 mg/ml R-spondin1, 100 ng/ml noggin [Peprotech], 100 ng/ml fibroblast growth factor 10 [Peprotech] and 100 ng/ml Wnt3A [R&D Systems, Minneapolis, MN, USA]) without using any feeder cells as described [22]. To exclude the effect of bacterial/fungal contamination to the organoid phenotype, we used Penicillin-streptomycin (100×; Invitrogen, cat. no. 15140–122) in organoid growth media. In the cytokine experiments, after isolating organoids from uninfected wild-type mice stomachs, we added either tumor necrosis factor (TNF)-α (10 ng/ml) or interleukin (IL)-1β (10 ng/ml) to serum-free organoid culture media, and measured the size of the organoids and mRNA expression levels. When we measured the organoids’ size, we viewed 10 fields of each well by the original magnification of 100X. 3 replicates was performed per group, and the software we used was EVOS®XL Core Cell Imaging System (ThermoFisher Scientific) and Microsoft Excel to calculate the average number and size of organoids.

### RNA extraction and cRNA microarray analysis

After culturing organoids from mouse gastric corpus infected with *H. felis* for 12 months or uninfected control, total cellular RNA was extracted from organoid using an acid guanidiumthiocyanate-phenol-chloroform method (ISOGEN Reagents; Nippon Gene, Tokyo, Japan) and column chromatography (RNeasy; Qiagen, Tokyo, Japan), according to the manufacturers’ instructions. Four RNA samples isolated from organoid, two experimental samples originated from mice infected with *H. felis* and two uninfected mice were used. For microarray analysis, cRNA mouse gene expression microarrays containing 39,430 Entrez Gene RNAs and 16,251 lincRNAs were used according to the manufacturer’s instructions. Genes were considered to be significantly upregulated in organoids infected with *H. felis* compared to control when they were upregulated in both arrays with an average log2 ratio exceeding 1 (two-fold). The data were confirmed by quantitative real-time polymerase chain reaction (qRT-PCR) analysis using primers (sequences were listed in Table 1).

**Table 1:** Primer sequences used in this study

Gene name	Forward	Reverse
*Gapdh*	GAC ATC AAG GTG AAG CAG	ATA CCA GGA AAT GAG CTT GAC AAA
*FlaB*	TTC GAT TGG TCC TAC AGG CTC AGA	TTC TTG ATG ACA TTG ACC AAC GCA
*mIl-1a*	GAG CGC TCA AGG AGA CCA G	CAG GTG CAC CCG ACT TTG TTC T
*mVillin*	GAC GTT TTC ACT GCC AAT ACC A	CCC AAG GCC CTA GTG AAG TCT T
*mMuc4*	GCT GCC TGT ATT CTT GCC T	ATG TTC TGG TGC TGG A
*mCd44*	TTG GTC ATA AAA GGG CTC TGT CAA	ATC ACC ACT ATG GCA AGC AAT GTC
*mDclk1*	GGG AGC CGA TTG TGT TTC AGA A	CTG TTG CCG TCC TGG AGT GTA A
*mIl-6*	CCG GAG AGG AGA CTT CAC AGA G	CTG CAA GTG CAT CGT TGT T
*mIl-1beta*	CAA GCA ACG ACA AAA TAC CTG TG	AGA CAA ACC GTT TTT CCA TCT
*mTnf-alpha*	TGG CCC AGA CCC TCA CAC TCA G	ACC CAT CGG CTG GCA CCA CT

### Immunohistochemical analysis

The following antibodies, listed with the catalog number, company, and dilution, were used for immunohistochemical analysis: TFF2 (sc-15,334, Santa Cruz, 1:100), MUC2 (orb13709, Biorbyt, 1:100), DCLK1 (AP7219b, Abgent, 1:100), VILLIN (sc-7672, Santa Cruz, 1:100), CK19 (ab15463, abcam, 1:50), MUC4 (sc-33,654, Santa Cruz, 1:100), CD44v6 (MCA1967, Serotec, 1:100). Immunohistochemical staining was performed on 4 μm sections with avidin-biotin-peroxidase complex kits (Vector Laboratories, USA) and counterstained with hematoxylin. For each mouse, 5 well-oriented gastric glands were scored to quantify the number of positive cells per gland. Data were expressed as average number of positive cells +/− S.D. in each group. Glyceraldehyde 3-phosphate dehydrogenase expression was used as an internal control (GAPDH, Cell Signaling, 2118, 1:1000).

### Xenograft tumor model

We first conducted MNU study in male C57BL6/J to generate organoids prior to subcutaneous injection into female NOD/SCID mice. Gastric epithelial cells were isolated and used for organoid culture. After 4 days of culture in Matrigel, we digested Matrigel with Cell Recovery Solution (Corning™ Cat No.354253), collect and centrifuge organoids, and re-suspended in PBS before injection. We injected the organoids subcutaneously into the flanks of 6-week-old female NOD/SCID mice. The sizes of the resulting tumors were measured using a caliper after sacrificing the mice. The tumors were isolated, paraffin-fixed, formalin-embedded, and subjected to histological analysis.

### Statistical analysis

Data were expressed as means ± standard error (SE). Differences were analyzed by non-parametric Mann-Whitney U test. A *p* value less than 0.05 were deemed to indicate statistical significance.

## Results

### The number of tissue stem/progenitor cells was increased in mouse stomach infected with *H. felis*

To assess the effect of chronic inflammation on the number of tissue stem/progenitor cells, we used a mouse gastritis model infected with *H. felis* for up to 12 months (Model 1 in Fig. 1). Histological analysis revealed severe gastric inflammation with lymphoid follicles, neck cell hyperplasia, oxyntic atrophy, and mucous metaplasia (SPEM), particularly in the gastric corpus, as described previously (Fig. 1b) [34]. We performed immunohistochemical analysis of TFF2, MUC2, CD44, DCLK1, and VILLIN, all of which have been proposed to be a tissue stem cell marker in gastrointestinal organs [20, 23, 35]. The expression levels of TFF2, MUC2, CD44, DCLK1, and VILLIN in *H. felis*-infected mice were significantly higher than those in uninfected control mice (Fig. 1b, c). Most of the cells positive for tissue stem cell markers were likely to be TFF2-positive, suggesting that certain cells in SPEM acquired stem cell properties. Those cells in SPEM also showed a proliferative marker, Ki67-positive (data not shown). These results indicated that chronic inflammation induced by *H. felis* infection affected the tissue stem cell property, which could be associated with gastric carcinogenesis.

**Fig. 2:**
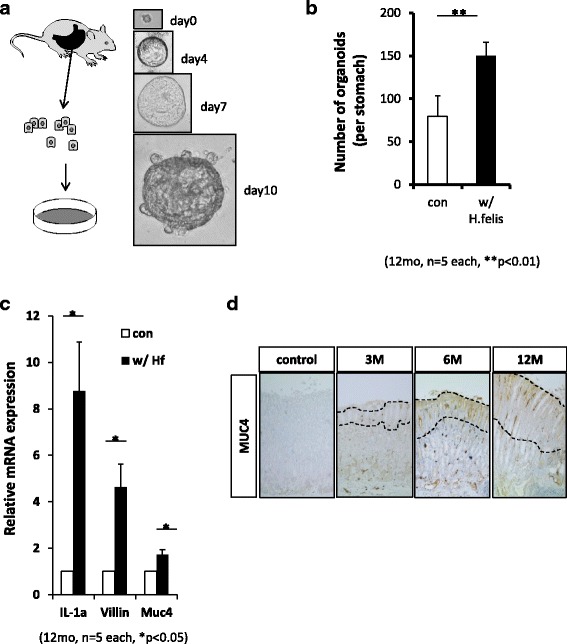
Characterization of gastric organoids established from *Helicobacter*-infected mouse stomachs. **a** Scheme and typical features of gastric organoid isolation from mouse gastric corpus. **b** The number of gastric organoids isolated from mouse gastric corpus, either uninfected or *H. felis* infected (*n* = 5 each group). **c** *IL-1α, Villin* and *Muc4* mRNA expression profiles in organoids isolated from mice gastric corpus infected with *H. felis* or uninfected (*n* = 5 each group)*.* **d** MUC4 protein in gastric mucosa infected with *H. felis* was detected by immunohistochemistry (original magnification ×200)

To assess the change in gastric epithelial cells in the stomach after chronic inflammation, we utilized 3D organoid culture systems using cells isolated from mice infected with *H. felis*, or uninfected controls, as described in Methods. Gastric organoids grew slowly and started to show budding at approximately 10 days after seeding (Fig. 2a). The organoids showed a single-layered epithelial structure under confocal microscopy with E-cadherin immunocytochemistry (data not shown). In accordance with the increased tissue stem/progenitor cells, the number of organoids cultured from *H. felis*-infected gastric epithelial cells was significantly higher than those from uninfected mice (Fig. 2b). we used half of the stomach for each mouse to establish the organoids. In this experiments, we used five same aged- male mice to generate the organoids.

### mRNA expression profile of organoids established from *Helicobacter*-infected mouse stomachs

To characterize the organoids in *H. felis*-infected mouse stomachs, we analyzed the mRNA expression profiles of organoids of the two groups using microarray and qRT-PCR. Among approximately 60,000 spots, 197 genes were up-regulated by two-fold or greater, and 581 genes were down-regulated less than 0.5 times. The top 50 upregulated or downregulated genes are listed in Tables 2, Additional file 1: Table S1 and Table S2. Among these significantly altered genes, intestinal-related genes, such as *Isx, Muc4, Muc16* and *Villin*, were upregulated, whereas gastric-related genes, such as Pepsinogen C and Gif were downregulated, demonstrating that *H. felis* infection caused intestinal alteration in gastric tissue stem/progenitor cells in vivo. Among the upregulated genes, we focused on genes reported to be associated with gastric carcinogenesis or genes known as stem cell marker, and confirmed that *interleukin-1α*, *Villin* and *Muc4* expression levels were increased in organoids isolated from *H. felis*-infected mice compared with control mice by real-time qRT-PCR analysis. (Fig. 2c, Table 2).

**Table 2:** Primer sequences used in this study

Gene name	Forward	Reverse
*Gapdh*	GAC ATC AAG GTG AAG CAG	ATA CCA GGA AAT GAG CTT GAC AAA
*FlaB*	TTC GAT TGG TCC TAC AGG CTC AGA	TTC TTG ATG ACA TTG ACC AAC GCA
*mIl-1a*	GAG CGC TCA AGG AGA CCA G	CAG GTG CAC CCG ACT TTG TTC T
*mVillin*	GAC GTT TTC ACT GCC AAT ACC A	CCC AAG GCC CTA GTG AAG TCT T
*mMuc4*	GCT GCC TGT ATT CTT GCC T	ATG TTC TGG TGC TGG A
*mCd44*	TTG GTC ATA AAA GGG CTC TGT CAA	ATC ACC ACT ATG GCA AGC AAT GTC
*mDclk1*	GGG AGC CGA TTG TGT TTC AGA A	CTG TTG CCG TCC TGG AGT GTA A
*mIl-6*	CCG GAG AGG AGA CTT CAC AGA G	CTG CAA GTG CAT CGT TGT T
*mIl-1beta*	CAA GCA ACG ACA AAA TAC CTG TG	AGA CAA ACC GTT TTT CCA TCT
*mTnf-alpha*	TGG CCC AGA CCC TCA CAC TCA G	ACC CAT CGG CTG GCA CCA CT

Muc4, an intestinal mucin, has been shown to be associated with gastric carcinogenesis [36–39]. Because *Muc4* was upregulated in organoids obtained from *H. felis*-infected mice, we performed immunohistochemistry analysis to confirm whether MUC4 was expressed in mouse stomachs infected with *H. felis*. MUC4 protein was expressed in gastric mucosa after 3 months of *H. felis* infection; furthermore, the area of MUC4-positive mucosa expanded as infection continued (Fig. 2d).

### Eradication of *H. felis* alters mRNA expression profile in organoids

**Fig. 3:**
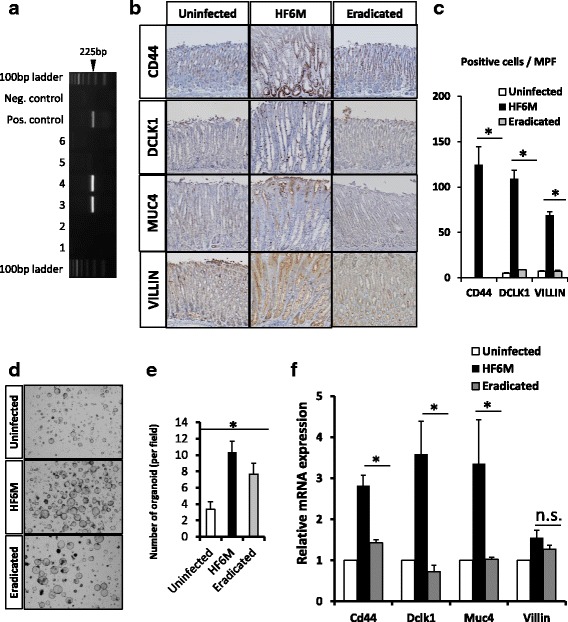
Eradication of *Helicobacter* infection alters the gastric tissue stem/progenitor cell marker profile. **a** Confirmation of infection status of *H. felis*. *FlaB* gene was amplified by PCR, and evaluated by gel electrophoresis. Lanes1,2; uninfected, lanes 3,4; infected, and lanes 5,6; eradicated mice. **b**, **c** Immunohistochemistry for tissue stem/progenitor cell markers. The number of cells positive for VILLIN, MUC4, and DCLK1 decreased after *H. felis* eradication. **d**, **e** Representative photographs of organoid from each group and the number of organoid in different status of *H. felis* infection (4 days after isolation, graph bars showed the average number of organoid in high power field. Values were average +/− S.E.). **f** mRNA expression profiles of tissue stem/progenitor cell markers in organoid

Since early eradication of *Helicobacter* inhibits gastric cancer progression [31], we analyzed the effect of *H. felis* eradication on the gene expression profile of gastric organoids. Eradication of *H. felis* was confirmed by *flaB* gene expression in gastric tissue at sacrifice (Fig. 3a). The mice were sacrificed and the stomachs were used for immunohistochemical analysis or isolation for 3D–organoid culture as described above. The protein expression of candidate gastric cancer stem/progenitor cells was assessed by using immunohistochemistry. Among the markers analyzed, the number of cells positive for *Cd44, Dclk1, Muc4,* or *Villin* was significantly reduced after eradication of *H. felis* (Fig. 3b). The number of organoids was also significantly reduced after *H. felis* eradication whereas the size of organoid was not decreased after *H. felis* eradication (Fig. 3c). We also assessed the mRNA expression of the stem cell markers above in organoid, and found that after *H. felis* eradication, all markers except *Villin*, were significantly down-regulated (Fig. 3d), suggesting that *H. felis*-induced SPEM or intestinal phenotype were reversible by the eradication of *H. felis*.

### Inflammatory cytokines induced the expression of stem cell markers

**Fig. 4:**
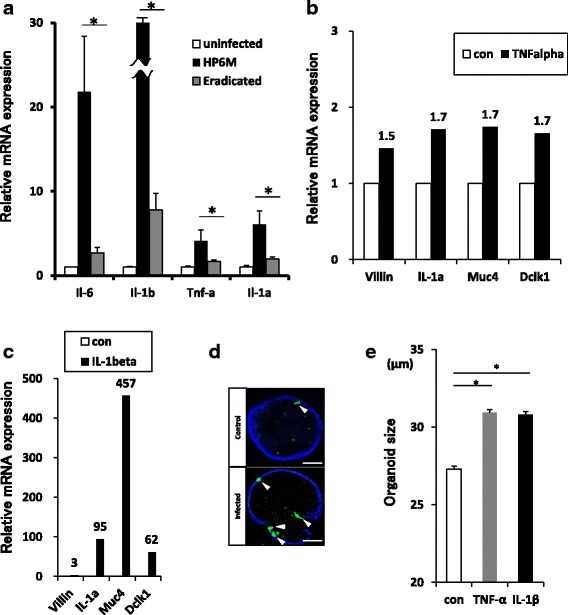
Cytokine profile of organoids isolated from mice stomach. **a** Cytokine profile of organoids isolated from uninfected, *H. felis*-infected, or eradicated mice gastric corpus. Values were relative mRNA expression +/− S.E. **b**, **c** mRNA expression profiles in wild-type mice gastric organoids 6 h after stimulation with cytokines. mRNA expression of putative tissue stem cell markers was increased in organoids after stimulation with pro-inflammatory cytokines. **d** Confocal micrograph of mouse gastric organoids. Immunocytochemical staining using an antibody against the putative tissue stem cell marker DCLK1 (green). 4′,6-Diamidino-2-phenylindole was used for counterstaining of nuclei. Scale bar: 20 μm. **e** The size of organoids after stimulation with each cytokine for 3 days

Chronic inflammation induced by *Helicobacter* infection is considered as a key factor in gastric carcinogenesis [40]. When we measured inflammatory cytokines in the organoids generated from each mouse before and after *H. felis* eradication, all four pro-inflammatory cytokines were increased in the organoid isolated from *H. felis* infected mice, and downregulated after eradication of *H. felis* (Fig. 4a). To further analyze the effect of inflammatory cytokines on gastric tissue stem/progenitor cells, we used the organoid culture system to determine whether inflammatory cytokines directly affected the phenotype or mRNA expression profile of gastric organoids. The mRNA levels of *Villin, IL-1α, Muc4,* and *Dclk1* were upregulated in organoids stimulated with inflammatory cytokines compared with untreated organoids. TNF-α administration demonstrated modest up-regulation of those four genes (Fig. 4b). On the other hands, stimulation with IL-1β resulted in marked upregulation of these genes (Fig. 4c). Immunostaining of organoids with an antibody against DCLK1 demonstrated that the number of DCLK1-positive cells was increased in organoids obtained from *H. felis*-infected stomachs (Fig. 4d). In addition, administration of each cytokine increased the size of organoid (Fig. 4e), suggesting that cytokine stimulation may play a role in inducing a genetic throwback from matured cell to the tissue stem/progenitor cell phenotype through the induction of stem/progenitor cell associated genes, which in turn accelerating their proliferation [41–44].

### Tumorigenic properties of organoids isolated from mouse stomachs

Finally, we assessed the tumorigenic property of organoids cultured from stomach using NOD/SCID mice. At day 4 of organoid culture, the organoids from MNU-treated mice showed early proliferation of cells, and exhibited an irregular morphology compared with those isolated from *H. felis*-infected or uninfected mice (Additional file 1: Figure S1A). After 2 months of injection of organoids into NOD/SCID, only organoids from MNU-treated mice produced tumors, and no tumors developed with organoids obtained from mice infected with *H. felis* or control mice (Additional file 1: Figure S1B). We confirmed that these tumors contained epithelial cells by using CK19 immunohistochemical staining (Additional file 1: Figure S1C). These results suggested that chronic inflammation alone may not be sufficient to induce malignant transformation in gastric tissue stem/progenitor cells, and other factors such as additional genetic/epigenetic changes in tissue stem/progenitor cells would be required for carcinogenesis.

## Discussion

In the present study, we demonstrated that chronic inflammation induced by *Helicobacter* infection caused the alteration in the mRNA/protein expression profile of organoids isolated from mouse stomachs. Muc4, an intestinal mucin protein, was upregulated in both gastric epithelium and organoids obtained from stomachs infected with *H. felis*. Moreover, expression levels of stem/progenitor cell markers such as *Cd44, Dclk1, Muc4* and *Villin* as well as cytokines were transiently increased after *H. felis* infection and decreased after the eradication of *H. felis*. Finally, we demonstrated that the organoids isolated from MNU-treated mice could develop tumors in NOD/SCID mice, whereas no tumors developed from control organoids.

Organotypic culture of the stomach and other tissue has been reported recently, particularly for regenerative medicine and cancer research [45, 46]. Although maintaining growth in primary cultures of epithelial cells is problematic [47], Barker et al. was successful using a 3D Matrigel culture technique with serum-free media. The advantage of organoid culture is analysis of cells without any contamination of non-epithelial cells such as inflammatory cells or stromal fibroblasts. Although tissue stem cell markers in the gastric corpus have not yet been elucidated, we and others have successfully established organoids from mouse stomachs infected with *Helicobacter* [48]. This technique enables recapitulation of the in vivo status of epithelial cells, enabling the investigation of the mechanisms underlying gastric atrophy, intestinal metaplasia, and carcinogenesis.

Chronic inflammation is known to play key roles in inflammation-associated cancer [40]. Long-term *Helicobacter* infection can cause oxyntic atrophy, intestinal metaplasia, and cancer; however, the precise mechanism by which inflammation causes cancer remains to be elucidated. Upon *Helicobacter* infection, gastric epithelial cells produce inflammatory cytokines, such as IL-8, and inflammatory cells recruited from the bone marrow accelerate the inflammatory reaction by producing TNF-α or IL-1β [34, 49, 50]. In the current study, we found that these cytokines were up-regulated in organoids obtained from stomachs infected with chronic *Helicobacter* infection. Furthermore, administration of these cytokines on organoids resulted in up-regulation of stem/progenitor cell markers, and promoted cell proliferation in vitro. These findings will strongly imply the connection between chronic inflammation and gastric carcinogenesis.

Many genes have been reported to be expressed in cancer-initiating cells, and targeting these cells could facilitate elimination of cancer cells [51]. *Cd44* is one of the most reliable marker for targeting gastric cancer [18], and elimination of CD44-positive cells using sulfasalazine plus cisplatin in patients with advanced gastric cancer has been attempted. In our data, *Cd44* mRNA was not detected in gastric corpus; however, in the antrum, it was increased in gastric epithelial cells in MNU-induced tumors. Targeting CD44-positive cells should be tested in gastric cancer animal model to evaluate its therapeutic potential for cancer.

Intestinal phenotype called SPEM in stomach has long time been considered as pre-cancerous lesion in gastric carcinogenesis [10]. In the current study, we found that many genes associated with intestinal phenotype were up-regulated in organoid isolated from *Helicobacter* infected gastric mucosa. In IHC staining, TFF2 and MUC2 were both positive in epithelial cells after chronic *Helicobacter* infection. Among those genes, Intestine specific homeobox gene, Isx, was up-regulated in *Helicobacter*-infected organoid. We have already reported that ISX could induce intestinal metaplasia and cell proliferation to contribute to gastric carcinogenesis [52]. In humans, eradication of *H. pylori* could lead the reduction of gastric carcinogenesis [6], however not all gastric cancer was eliminated after *H. pylori* eradication. Since Intestinal metaplasia has not been completely disappeared after *H. pylori* eradication, acquisition of stemness or metaplastic phenotype after chronic inflammation through the genetic/epigenetic changes in gastric tissue stem cells could have strong influence in gastric carcinogenesis [41]. Collectively, intestinal phenotype in stomach would be not just a differentiated metaplasia in stomach, but as a phenotype of stem cell abnormality with precancerous lesion susceptible to gastric carcinogenesis after chronic inflammation.

## Conclusions

In summary, we demonstrated here that, as one of the possible mechanisms of gastric carcinogenesis, chronic inflammation induced by *Helicobacter* infection can increase the number of tissue stem/progenitor cells, promote the proliferation of those cells, and alter the properties of stem cells toward intestinal metaplasia to cancer. An organoid culture system combined with a *Helicobacter*-infected gastric cancer model and xenograft model would enable to identify cancer-initiating cells and investigation of inflammation-associated gastric carcinogenesis. Using this organoid system with primary gastric cells from patients would serve as not only a tool for basic cancer research but also serve as a tool for drug screening in cancer therapy to implement personalized medicine in the future.

## Additional files

Additional file 1: Figure S1. Tumorigenic properties of organoids isolated from mouse stomachs. (A) Representative histological findings in MNU-treated stomachs, and the scheme of the subcutaneous tumor model in NOD/SCID mice. (B) Subcutaneous tumors in NOD/SCID mice. A palpable nodule (arrow) at 5 weeks post injection and immunohistochemistry of the tumor (anti-CK19). (C) Summary of tumor development in NOD/SCID mice at 5 weeks post infection. **Table S1.** List of the 50 genes significantly upregulated between organoids infected with *H. felis* and uninfected organoids. **Table S2.** List of the 50 genes significantly downregulated between organoids infected with *H. felis* and uninfected organoids. (PPTX 575 kb)

## Abbreviations

*H. felis*: *Helicobacter felis*
*H. pylori*: *Helicobacter pylori*;
IL: Interleukin
MNU: N-methyl-N-nitroso-urea
PBS: phosphate-buffered saline
qRT-PCR: quantitative real-time polymerase chain reaction
SE: Standard error
SPEM: Spasmolytic polypeptide-expressing metaplasia
TFF: Trefoil factor
TNF: Tumor necrosis factor
