# Correction to: *Helicobacter*-induced gastric inflammation alters the properties of gastric tissue stem/progenitor cells

**DOI:** 10.1186/s12876-017-0733-3

**Published:** 2018-01-08

**Authors:** Wataru Shibata, Soichiro Sue, Sachiko Tsumura, Yasuaki Ishii, Takeshi Sato, Eri Kameta, Makoto Sugimori, Hiroaki Yamada, Hiroaki Kaneko, Tomohiko Sasaki, Tomohiro Ishii, Toshihide Tamura, Masaaki Kondo, Shin Maeda

**Affiliations:** 1 Department of Gastroenterology, Yokohama City University Graduate School of Medicine, Yokohama, Japan; 2 Division of Translational Research, Advanced Medical Research Center, Yokohama City University, Yokohama, Japan; 3 School of Medicine, Yokohama City University, Yokohama, Japan

## Correction

Unfortunately, the original article [1] contained an error incorporated during production. A duplicated version of Table 1 was published in place of Table 2. Table 2 has been corrected in the original article and is also included correctly below.

**Table 2:** List of representative genes differentially expressed between organoids with and without *H. felis* infection

Gene Name	Log2 ratio
Intestine related genes	
*Isx*	2.75
*Muc4*	2.10
*Muc16*	1.70
*Vil1*	1.10
Gastric genes	
*Pepsinogen C*	− 3.17
*Gif*	− 2.47
Others	
*Gastrin*	3.30
*Cd34*	2.77
*Il-1a*	2.51
*Cd274 (Pd-l1)*	1.31
