# Supplementary material for: Helicobacter-induced gastric inflammation alters the properties of gastric tissue stem/progenitor cells
Source: BMC Gastroenterol. 2017 Dec 6;17:145. doi: 10.1186/s12876-017-0706-6 (PMC5719643; doi:10.1186/s12876-017-0706-6)
Supplement: Supplementary file 1 — Tumorigenic properties of organoids isolated from mouse stomachs. (A) Representative histological findings in MNU-treated stomachs, and the scheme of the subcutaneous tumor model in NOD/SCID mice. (B) Subcutaneous tumors in NOD/SCID mice. A palpable nodule (arrow) at 5 weeks post injection and immunohistochemistry of the tumor (anti-CK19). (C) Summary of tumor development in NOD/SCID mice at 5 weeks post infection. Table S1. List of the 50 genes significantly upregulated between organoids infected with H. felis and uninfected organoids. Table S2. List of the 50 genes significantly downregulated between organoids infected with H. felis and uninfected organoids. (PPTX 575 kb) [file 12876_2017_706_MOESM1_ESM.pptx]

## Slide 1
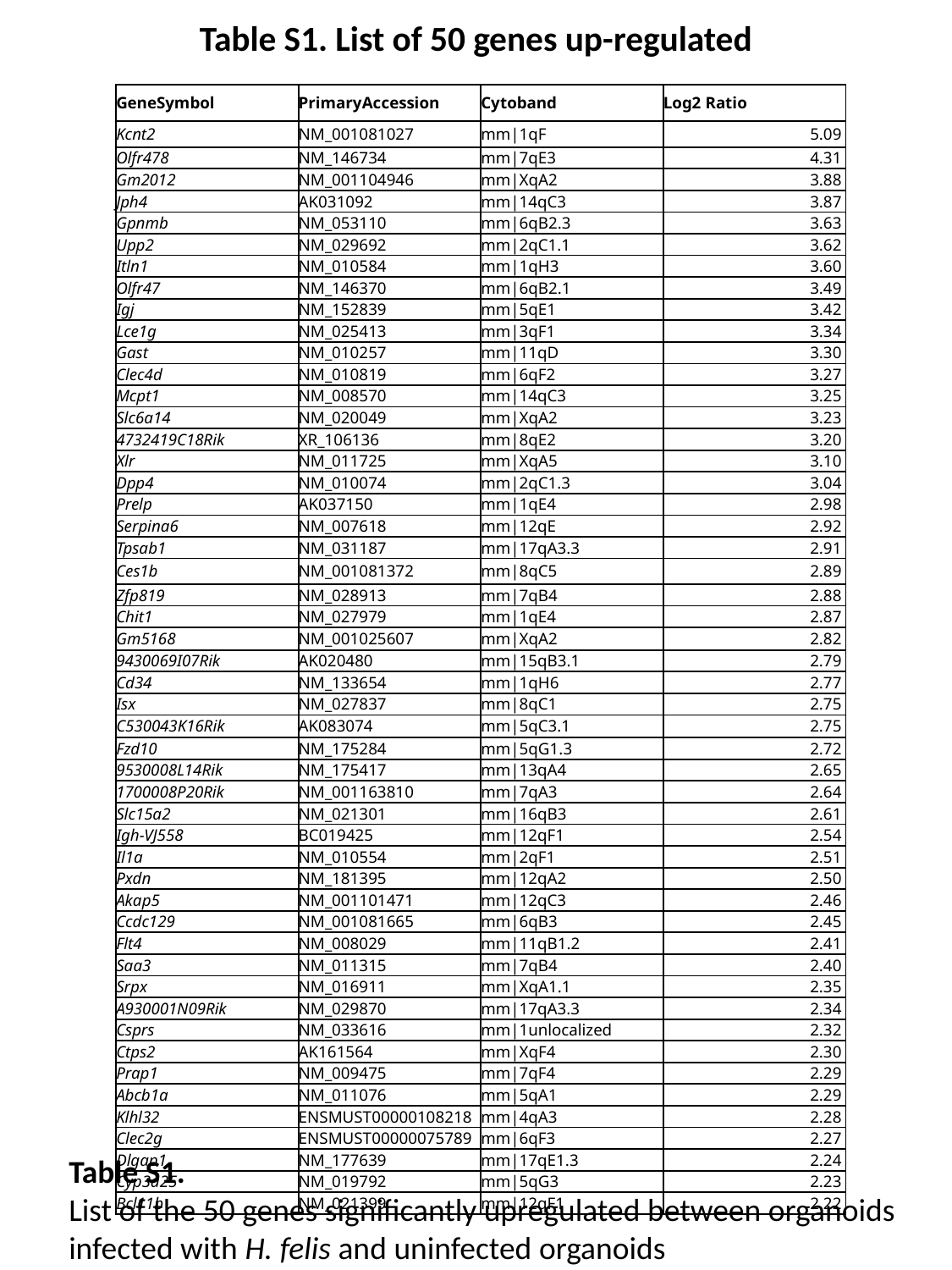

# Table S1. List of 50 genes up-regulated
| GeneSymbol | PrimaryAccession | Cytoband | Log2 Ratio |
| --- | --- | --- | --- |
| Kcnt2 | NM\_001081027 | mm|1qF | 5.09 |
| Olfr478 | NM\_146734 | mm|7qE3 | 4.31 |
| Gm2012 | NM\_001104946 | mm|XqA2 | 3.88 |
| Jph4 | AK031092 | mm|14qC3 | 3.87 |
| Gpnmb | NM\_053110 | mm|6qB2.3 | 3.63 |
| Upp2 | NM\_029692 | mm|2qC1.1 | 3.62 |
| Itln1 | NM\_010584 | mm|1qH3 | 3.60 |
| Olfr47 | NM\_146370 | mm|6qB2.1 | 3.49 |
| Igj | NM\_152839 | mm|5qE1 | 3.42 |
| Lce1g | NM\_025413 | mm|3qF1 | 3.34 |
| Gast | NM\_010257 | mm|11qD | 3.30 |
| Clec4d | NM\_010819 | mm|6qF2 | 3.27 |
| Mcpt1 | NM\_008570 | mm|14qC3 | 3.25 |
| Slc6a14 | NM\_020049 | mm|XqA2 | 3.23 |
| 4732419C18Rik | XR\_106136 | mm|8qE2 | 3.20 |
| Xlr | NM\_011725 | mm|XqA5 | 3.10 |
| Dpp4 | NM\_010074 | mm|2qC1.3 | 3.04 |
| Prelp | AK037150 | mm|1qE4 | 2.98 |
| Serpina6 | NM\_007618 | mm|12qE | 2.92 |
| Tpsab1 | NM\_031187 | mm|17qA3.3 | 2.91 |
| Ces1b | NM\_001081372 | mm|8qC5 | 2.89 |
| Zfp819 | NM\_028913 | mm|7qB4 | 2.88 |
| Chit1 | NM\_027979 | mm|1qE4 | 2.87 |
| Gm5168 | NM\_001025607 | mm|XqA2 | 2.82 |
| 9430069I07Rik | AK020480 | mm|15qB3.1 | 2.79 |
| Cd34 | NM\_133654 | mm|1qH6 | 2.77 |
| Isx | NM\_027837 | mm|8qC1 | 2.75 |
| C530043K16Rik | AK083074 | mm|5qC3.1 | 2.75 |
| Fzd10 | NM\_175284 | mm|5qG1.3 | 2.72 |
| 9530008L14Rik | NM\_175417 | mm|13qA4 | 2.65 |
| 1700008P20Rik | NM\_001163810 | mm|7qA3 | 2.64 |
| Slc15a2 | NM\_021301 | mm|16qB3 | 2.61 |
| Igh-VJ558 | BC019425 | mm|12qF1 | 2.54 |
| Il1a | NM\_010554 | mm|2qF1 | 2.51 |
| Pxdn | NM\_181395 | mm|12qA2 | 2.50 |
| Akap5 | NM\_001101471 | mm|12qC3 | 2.46 |
| Ccdc129 | NM\_001081665 | mm|6qB3 | 2.45 |
| Flt4 | NM\_008029 | mm|11qB1.2 | 2.41 |
| Saa3 | NM\_011315 | mm|7qB4 | 2.40 |
| Srpx | NM\_016911 | mm|XqA1.1 | 2.35 |
| A930001N09Rik | NM\_029870 | mm|17qA3.3 | 2.34 |
| Csprs | NM\_033616 | mm|1unlocalized | 2.32 |
| Ctps2 | AK161564 | mm|XqF4 | 2.30 |
| Prap1 | NM\_009475 | mm|7qF4 | 2.29 |
| Abcb1a | NM\_011076 | mm|5qA1 | 2.29 |
| Klhl32 | ENSMUST00000108218 | mm|4qA3 | 2.28 |
| Clec2g | ENSMUST00000075789 | mm|6qF3 | 2.27 |
| Dlgap1 | NM\_177639 | mm|17qE1.3 | 2.24 |
| Cyp3a25 | NM\_019792 | mm|5qG3 | 2.23 |
| Bcl11b | NM\_021399 | mm|12qF1 | 2.22 |
Table S1.
List of the 50 genes significantly upregulated between organoids infected with H. felis and uninfected organoids

## Slide 2
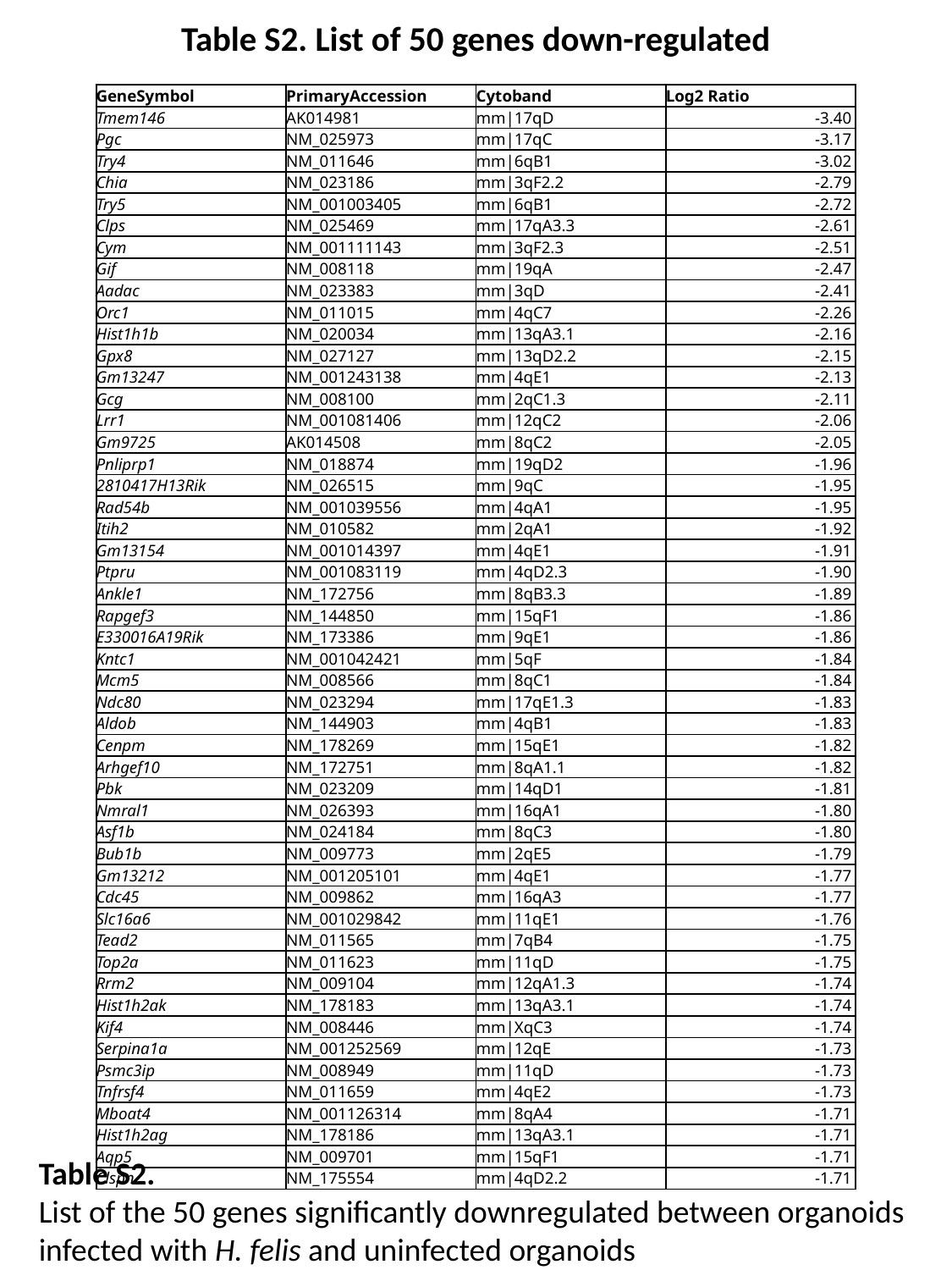

# Table S2. List of 50 genes down-regulated
| GeneSymbol | PrimaryAccession | Cytoband | Log2 Ratio |
| --- | --- | --- | --- |
| Tmem146 | AK014981 | mm|17qD | -3.40 |
| Pgc | NM\_025973 | mm|17qC | -3.17 |
| Try4 | NM\_011646 | mm|6qB1 | -3.02 |
| Chia | NM\_023186 | mm|3qF2.2 | -2.79 |
| Try5 | NM\_001003405 | mm|6qB1 | -2.72 |
| Clps | NM\_025469 | mm|17qA3.3 | -2.61 |
| Cym | NM\_001111143 | mm|3qF2.3 | -2.51 |
| Gif | NM\_008118 | mm|19qA | -2.47 |
| Aadac | NM\_023383 | mm|3qD | -2.41 |
| Orc1 | NM\_011015 | mm|4qC7 | -2.26 |
| Hist1h1b | NM\_020034 | mm|13qA3.1 | -2.16 |
| Gpx8 | NM\_027127 | mm|13qD2.2 | -2.15 |
| Gm13247 | NM\_001243138 | mm|4qE1 | -2.13 |
| Gcg | NM\_008100 | mm|2qC1.3 | -2.11 |
| Lrr1 | NM\_001081406 | mm|12qC2 | -2.06 |
| Gm9725 | AK014508 | mm|8qC2 | -2.05 |
| Pnliprp1 | NM\_018874 | mm|19qD2 | -1.96 |
| 2810417H13Rik | NM\_026515 | mm|9qC | -1.95 |
| Rad54b | NM\_001039556 | mm|4qA1 | -1.95 |
| Itih2 | NM\_010582 | mm|2qA1 | -1.92 |
| Gm13154 | NM\_001014397 | mm|4qE1 | -1.91 |
| Ptpru | NM\_001083119 | mm|4qD2.3 | -1.90 |
| Ankle1 | NM\_172756 | mm|8qB3.3 | -1.89 |
| Rapgef3 | NM\_144850 | mm|15qF1 | -1.86 |
| E330016A19Rik | NM\_173386 | mm|9qE1 | -1.86 |
| Kntc1 | NM\_001042421 | mm|5qF | -1.84 |
| Mcm5 | NM\_008566 | mm|8qC1 | -1.84 |
| Ndc80 | NM\_023294 | mm|17qE1.3 | -1.83 |
| Aldob | NM\_144903 | mm|4qB1 | -1.83 |
| Cenpm | NM\_178269 | mm|15qE1 | -1.82 |
| Arhgef10 | NM\_172751 | mm|8qA1.1 | -1.82 |
| Pbk | NM\_023209 | mm|14qD1 | -1.81 |
| Nmral1 | NM\_026393 | mm|16qA1 | -1.80 |
| Asf1b | NM\_024184 | mm|8qC3 | -1.80 |
| Bub1b | NM\_009773 | mm|2qE5 | -1.79 |
| Gm13212 | NM\_001205101 | mm|4qE1 | -1.77 |
| Cdc45 | NM\_009862 | mm|16qA3 | -1.77 |
| Slc16a6 | NM\_001029842 | mm|11qE1 | -1.76 |
| Tead2 | NM\_011565 | mm|7qB4 | -1.75 |
| Top2a | NM\_011623 | mm|11qD | -1.75 |
| Rrm2 | NM\_009104 | mm|12qA1.3 | -1.74 |
| Hist1h2ak | NM\_178183 | mm|13qA3.1 | -1.74 |
| Kif4 | NM\_008446 | mm|XqC3 | -1.74 |
| Serpina1a | NM\_001252569 | mm|12qE | -1.73 |
| Psmc3ip | NM\_008949 | mm|11qD | -1.73 |
| Tnfrsf4 | NM\_011659 | mm|4qE2 | -1.73 |
| Mboat4 | NM\_001126314 | mm|8qA4 | -1.71 |
| Hist1h2ag | NM\_178186 | mm|13qA3.1 | -1.71 |
| Aqp5 | NM\_009701 | mm|15qF1 | -1.71 |
| Clspn | NM\_175554 | mm|4qD2.2 | -1.71 |
Table S2.
List of the 50 genes significantly downregulated between organoids infected with H. felis and uninfected organoids

## Slide 3
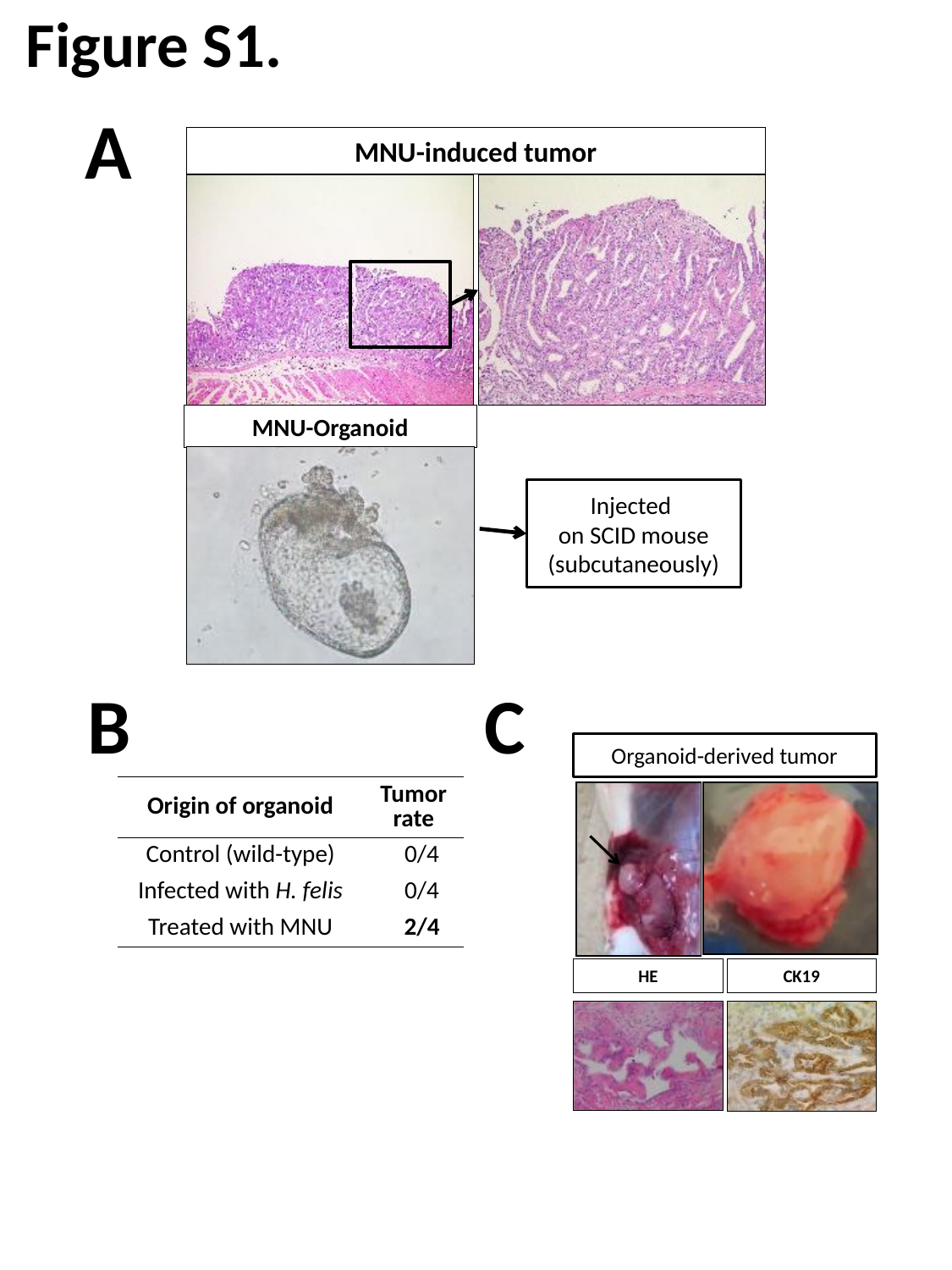

Figure S1.
A
MNU-induced tumor
CD44
MNU-Organoid
Injected
on SCID mouse (subcutaneously)
B
C
Organoid-derived tumor
| Origin of organoid | Tumor rate |
| --- | --- |
| Control (wild-type) | 0/4 |
| Infected with H. felis | 0/4 |
| Treated with MNU | 2/4 |
HE
CK19
